# Thermodynamic stability and optoelectronic property modulation of KTaO_3_ via substitutional doping with Sr, Ba, La and Pr

**DOI:** 10.1038/s41598-026-49310-8

**Published:** 2026-04-21

**Authors:** Farrukh Javed, Syed Muhammad Alay-e-Abbas, Akram Ibrahim, Ghulam Abbas

**Affiliations:** 1 Computational Materials Modeling Laboratory, Department of Physics, Government College University, Faisalabad, Faisalabad, 38040 Pakistan; 2 Department of Physics, College of Science, King Khalid University, P. O. Box: 9004, Abha, 61413 Asir Kingdom of Saudi Arabia; 3 Department of Physics, Chemistry and Biology, Linköping University, Linköping, 58183 Sweden

**Keywords:** Perovskite oxides, Rare-earth dopants, Thermodynamic stability, Electronic structure, Optical properties, Materials science, Physics

## Abstract

In this work, we employ density functional theory (DFT) calculations to investigate the thermodynamics and optoelectronic properties of alkaline-earth (Sr and Ba) and rare-earth (La and Pr) metal dopants when introduced as substitutional defects in KTaO_3_. The structural, energetic and thermodynamic stability of the doped systems are examined with SCAN meta-GGA functional. On the other hand, the mBJ-LDA meta-GGA is employed for more accurate description of electronic and optical properties. Our results indicate that Sr, Ba and La dopants preferably substitute at the K-site of KTaO_3_. In contrast, Pr doping is thermodynamically more stable at the Ta-site of KTaO_3_. While Sr-, Ba- and La-doped systems exhibit electron-doped semiconductor behaviour, their threshold of optical absorption shift towards higher energy UV photons. On the contrary, doping of Pr at a Ta-site of KTaO_3_ is found to be stable under oxygen-rich chemical environment and leads to a hole-doped semiconductor behaviour with optical absorption threshold shifted towards the visible region. Owing to growing interest in the properties emerging from doping of alkaline-earth and rare-earth elements in KTaO_3_ in recent experiments, the outcomes of this study provide valuable insights into designing materials suitable for photocatalytic applications.

## Introduction

In the past couple of decades, photocatalysis has gained considerable attention from scientific community owing to its potential in resolving the energy and environment related problems^1,2^. Although various techniques have been employed to remove pollutants from water and to break bonds between hydrogen and oxygen in water molecule for H_2_ evolution, photocatalysis using solar energy appears to be an excellent way to achieve these processes owing to its commercial, social and environmental benefits^3^. However, the low quantum yield and degrading photocatalytic efficiency over time of the currently employed materials are the main obstacles behind their practical applications. For this reason, much efforts have been put into designing efficient photocatalytic materials.

Semiconductor oxides are widely studied because of their potential applications as photocatalysts active in the visible and UV region of the electromagnetic spectrum^4–6^. Among these oxide materials, TiO_2_ is ubiquitously employed in environmental applications due to its non-toxicity, high photocatalytic activity and chemical stability. Unfortunately, shortcomings like low solar efficiency restrict its practical applications. While numerous efforts have been expended for the improvement in the photocatalytic activity of TiO_2_, only minor progress has been made^7–12^. The ABO_3_ perovskites, where A and B are metal atoms, comprise a large family of materials with applications ranging from water splitting to energy harvesting^13–21^. They are sufficiently long lasting, nontoxic and mostly composed of naturally abundant elements. Most importantly, the optical properties of these materials can be tuned by introducing various foreign elements at the A, B and O sites of ABO_3_ perovskites. Among the perovskite oxides, alkali-metal tantalates are regarded as highly efficient photocatalysts for hydrogen production^22^. Studies conducted on ATaO_3_ (A = Li, Na and K) in the past three decades clearly show that tantalates have good photocatalytic properties^14,23–27^. Among these tantalates, KTaO_3_ (KTO) adopts the cubic perovskite structure and has an indirect band gap of 3.640 eV to 3.757 eV^28,29^. However, like other members of its family, the wide band gaps, low quantum efficiencies, optical absorption in the UV region and relatively high reduction potential result in low photocatalytic reaction rates for KTO^17,30^. Therefore, the application of pristine KTO as an efficient photocatalyst remains a challenge.

As the incorporation of foreign metal elements into the crystal structure of a pristine semiconductor allows for reducing its band gap, it can also provide opportunities for improving the photocatalytic performance of KTO^31–35^. Among the various elemental dopants available for incorporation at the K- and Ta-site of KTO for improving its photocatalytic properties, particular attention has been paid to alkaline-earth and rare-earth elements^36–43^. Doping of the alkaline-earth metals such as Sr has been recently examined, where simultaneous occupancy of the K-site and Ta-site has been reported^44^. The concentration gradient of Sr dopants from K-site to Ta-site was suggested to be responsible for an energy gradient of the conduction band edge bottom, which caused the photocatalytic activity of Sr-doped KTO to decrease^44^. However, other experimental studies suggest that the incorporation of alkaline-earth metal such as Ba at the K-site^45–47^ results in an electron doped system with good transport properties. This is particularly important in view of the fact that incorporation of La^3+^ at the K-site of KTO generates a more dense and dispersive band structure, which allows promotion and separation of delocalized electron-hole pairs for better photocatalytic activity^36^. Sudrajat *el al.* showed that La doping at the K-site of KTO causes an enhancement in the photocatalytic activity of KTaO_3_^37^. However, the band gap of Sr-, Ba- and La-doped system does not decrease and the photocatalysis is only possible in UV region of electromagnetic spectrum^36,37^. These recent studies therefore raise the questions that whether or not alkaline-earth and rare-earth metals are thermodynamically stable at the K- or Ta-site of KTO? And if yes, what impact do they have on the electronic structure of the doped system? To address these questions, we have resorted to density functional theory (DFT)^38^ which facilitates understanding of atomistic level changes in material properties emerging from substitutional doping. As our recent work on the alkali-metal tantalates shows that energetic, structural, mechanical, vibrational, electronic and optical properties can be reliably obtained by combining meta-GGA functionals of DFT^39^, in this work we employ the same strategy to explore the impact of Sr, Ba, La and Pr as dopants on the physical properties of KTO. The motivation for exploring properties of substitutional doping of Sr, Ba and La in KTO is evident from the above-cited experimental literature where *n*-type doping is shown to enhance the photocatalytic properties of KTO^37,44,46,47^. On the other hand, the choice of exploring doping feasibility of praseodymium in KTO is based on its partially filled *4f* orbitals that are expected to give rise to mid-gaps states in KTO^40^. Our calculations reveal that doping of Pr at the Ta-site of KTO can be stabilized under oxygen-rich chemical environments. Moreover, with a hole doped electronic structure, Pr-doped KTO is also found to exhibit improved optical absorption in the visible region of the electromagnetic spectrum. The results presented in this work can prove significant in identifying and designing future KTO based photocatalysts for hydrogen production.

## Computational method

All the theoretical calculations presented in this work were carried out using the full-potential linear augmented planewave (FP-LAPW) method as available in the WIEN2k software package^41^. The strongly constrained and appropriately normed (SCAN) meta-GGA functional of DFT was employed to compute the total energies and structural optimization^42^. As the SCAN functional normally predicts an underestimated band gap compared to experiment, the modified Becke-Jhonson local density approximation (mBJ-LDA) potential functional was also taken into consideration to accurately calculate the electronic and optical properties^43^. The muffin-tin radii were selected to be 1.9, 1.85, 1.6, 2.1, 2.3, 2.31 and 2.33 Bohr for the K, Ta, O, Sr, Ba, La and Pr atoms, respectively, while an energy cutoff of -6.0 Ry was selected to separate the core states from the valence states. The spin-orbit coupling (SOC) effects have been included for electronic and optical properties. In these calculations, the values of the Fourier expansion of the charge density vector (G_max_), plane wave cutoff (R_O_K_max_) and angular momentum were 24, 8 and 10 Bohr^− 1^, respectively.

Since the values of lattice parameters for the cubic unit cell of KTO predicted by the SCAN meta-GGA are in good agreement with the previously reported experimental and theoretical lattice parameters^48,49^, we use this cubic unit cell to prepare structural models of the doped systems studied in this work. The doped systems were created with a 40 atom 2 × 2 × 2 supercell of cubic KTO. In this work, we introduced the alkaline-earth metals (Sr and Ba) or lanthanide elements (La and Pr) at the K- and Ta-sites of the KTaO_3_ perovskite. Two configurations for each metal dopant have been generated by substitutional doping of alkaline-earth or rare-earth metals at the K- and Ta-sites of KTO, which are denoted by X_K_ and X_Ta_, respectively. In all the doped systems, Pm-3 m cubic symmetry is achieved within the 2 × 2 × 2 supercell for which total energy calculations were carried out by employing a 5* × *5* × *5 k-mesh. In this work, we consider the thermodynamic conditions where Sr, Ba, La and Pr dopants can be introduced into KTO without the presence of other competing dopants, such as oxygen vacancies. Moreover, no charge compensation procedure (e.g. background charges) is adopted in this work that can influence the *n*-type or *p*-type nature originating from the difference in the valence states of the dopant atom and K/Ta. For the compounds examined in this work, the self consistent calculations were performed iteratively until the energy difference between two successive self consistent cycles was less than 10^− 5^ Ry. In addition, the atomic positions inside the Sr, Ba, La and Pr-doped KTO supercells were completely relaxed until the force on each atom was less than 1 m Ry/a.u.

## Results and discussion

### Chemical stability diagram of KTaO_3_

**Fig. 1:**
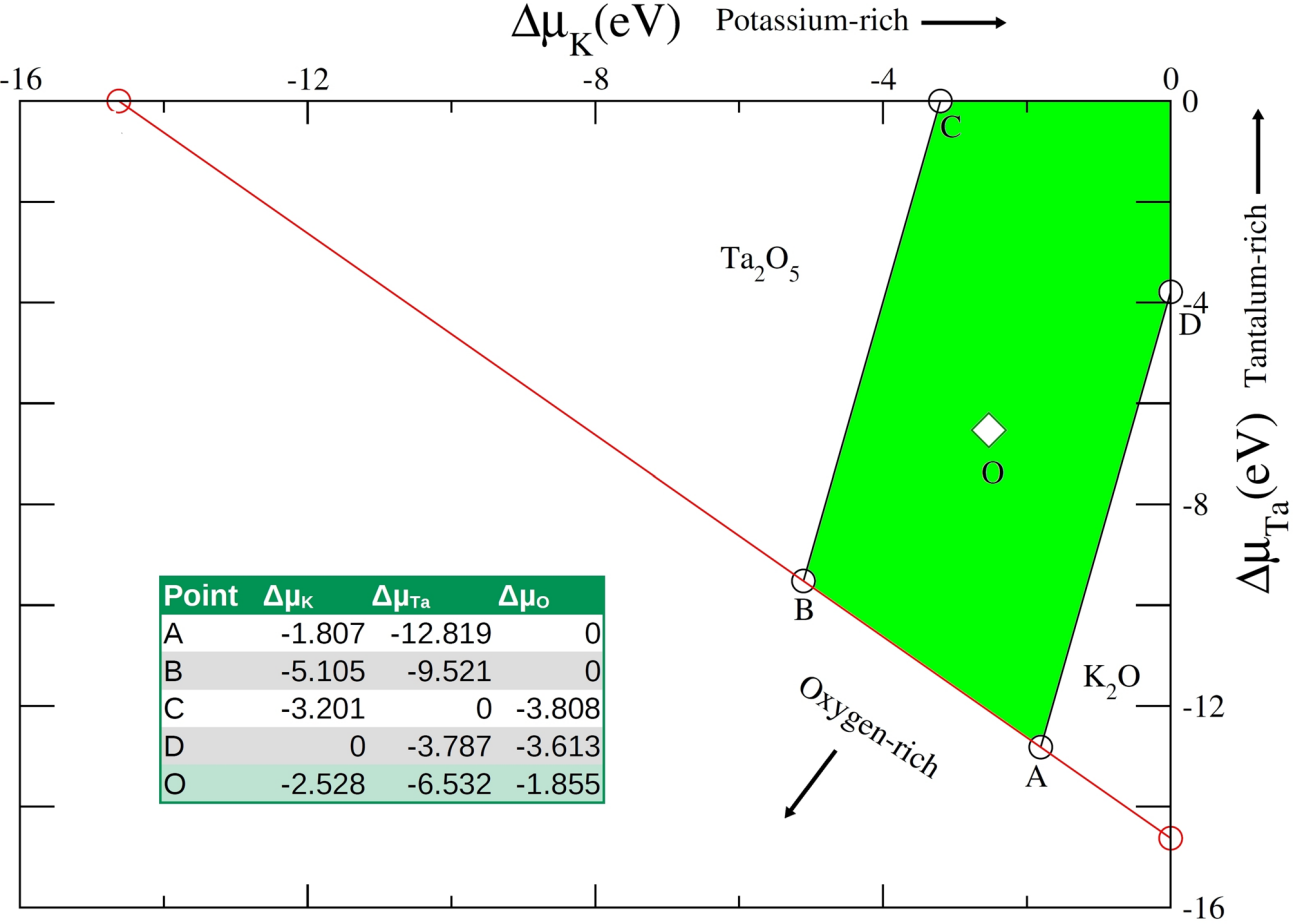
The stability diagram of pristine KTaO_3_ calculated using SCAN meta-GGA functional. Points A-B-C-D indicate the region where chemical potentials of K, Ta and O allow formation of KTaO_3_ without the presence of competing binary phases K_2_O and Ta_2_O_5_.

The stable formation of a bulk perovskite material and the incorporation of various doping elements in it depends on the thermodynamic stability region mapped out by the chemical potentials of the elemental species involved in the system^50,51^. Due to this, we first construct the stability region of KTO using the total atomic chemical potentials of K ($\:{\mu\:}_{K}={E}_{t}^{K}+{\varDelta\:\mu\:}_{K}$), Ta ($\:{\mu\:}_{Ta}={E}_{t}^{Ta}+{\varDelta\:\mu\:}_{Ta}$) and O ($\:{\mu\:}_{O}=\frac{1}{2}{E}_{t}^{{O}_{2}}+{\varDelta\:\mu\:}_{O}$) with reference to the their respective stable solid and gaseous phases^51^. This first requires that the variations of the atomic chemical potential *Δµ*_K_, *Δµ*_Ta_ and *Δµ*_O_ should not be greater than zero. Moreover, for KTO to be stable in the cubic perovskite structure, the variations in the atomic chemical potentials should satisfy the following equations to avoid synthesis of K_2_O and Ta_2_O_5_,

1 $$\:{\varDelta\:H}_{f}^{{KTaO}_{3}}={\varDelta\:\mu\:}_{K}+{\varDelta\:\mu\:}_{Ta}+3{\varDelta\:\mu\:}_{O}$$

2 $$\:{\varDelta\:H}_{f}^{{K}_{2}O}\ge\:2{\varDelta\:\mu\:}_{K}+{\varDelta\:\mu\:}_{O}$$

3 $$\:{\varDelta\:H}_{f}^{{Ta}_{2}{O}_{5}}\ge\:2{\varDelta\:\mu\:}_{Ta}+5{\varDelta\:\mu\:}_{O}$$

which are the competing binary oxides for pristine KTO. Using the enthalpies of formation of KTO ($\:{\varDelta\:H}_{f}^{{KTaO}_{3}}$=−14.626 eV/f.u.), K_2_O ($\:{\varDelta\:H}_{f}^{{K}_{2}O}$=−3.613 eV/f.u.) and Ta_2_O_5_ ($\:{\varDelta\:H}_{f}^{{Ta}_{2}{O}_{5}}$=−19.042 eV/f.u.)^39^, we have constructed the chemical stability diagram of pristine KTO, which is shown in Fig. 1. The extreme values of atomic chemical potentials of K, Ta and O are shown by the points A, B, C and D. It is important to note here that the equality in Eq. (2) requires that inequality in the Eq. (3) is true, which is satisfied at both points A and D. On the other hand, the equality in Eq. (3) requires that inequality in Eq. (2) is true, which is satisfied at both points B and C.

While the values of atomic chemical potentials residing within the green shaded area enclosed by the points A-B-C-D ensure stable synthesis of KTO, any set of {*Δµ*_K_, *Δµ*_Ta_, *Δµ*_O_} values with *Δµ*_K_ = 0 eV (i.e. the vertical axis in Fig. 1) and *Δµ*_Ta_ = 0 eV (i.e. the horizontal axis of Fig. 1) represent potassium-rich and tantalum-rich conditions, respectively. However, for stable production of KTO, the largest permissible chemical potential values for K and O under extreme tantalum-rich conditions (i.e. point C) are *Δµ*_*K*_ =−3.201 eV and *Δµ*_*O*_ =−3.808 eV, respectively. Since the most negative *Δµ*_*O*_ is also achieved at point C of the chemical stability diagram shown in Fig. 1, this point is also the oxygen-poor chemical environment. On the other hand, the line A-B at the bottom of the green shaded area in Fig. 1 represents the oxygen-rich chemical environment where *Δµ*_*O*_ = 0 eV. As the largest negative atomic chemical potential of Ta is possible at point A of stability diagram (where *Δµ*_*Ta*_ =−12.819 eV), this point represents the tantalum-poor condition. The center of mass of green shaded area is denoted by point O where the atomic chemical of Ta and O are in between their respective extreme values.

### Defect formation energies

In order to be able to reliably evaluate the influence of Sr, Ba, La and Pr on the physical properties and photocatalytic performance of KTaO_3_, it is important to first evaluate the relative stability of these dopants at the two possible substitutional sites (i.e. K-site and Ta-site of KTO). Therefore, the stability of each doped system in the two doping configurations was examined by employing the defect formation energy analysis. To evaluate the successful incorporation of the dopants considered in this work, it is also necessary that the selected atomic chemical potential of oxygen should prevent the formation of binary oxides (BaO, SrO, La_2_O_3_ and Pr_2_O_3_) as competing phases during the doping process. Therefore, we need to impose additional constraints on the atomic chemical potentials of atoms being added to and removed from KTO lattice. Equation (4) through (7) provide the relationship between the enthalpies of formation of SrO, BaO, La_2_O_3_ and Pr_2_O_3_ and the allowed atomic chemical potentials of oxygen within the chemical stability diagram of KTO, which were used for determining the atomic chemical potentials of Sr, Ba, La and Pr

4 $$\:{\varDelta\:\mu\:}_{Sr}={\varDelta\:H}_{f}^{SrO}-{\varDelta\:\mu\:}_{O}$$

5 $$\:{\varDelta\:\mu\:}_{Ba}={\varDelta\:H}_{f}^{BaO}-{\varDelta\:\mu\:}_{O}$$

6 $$\:{\varDelta\:\mu\:}_{La}=\frac{{\varDelta\:H}_{f}^{{La}_{2}{O}_{3}}-3{\varDelta\:\mu\:}_{O}}{2}$$

7 $$\:{\varDelta\:\mu\:}_{Pr}=\frac{{\varDelta\:H}_{f}^{{Pr}_{2}{O}_{3}}-3{\varDelta\:\mu\:}_{O}}{2}$$

**Fig. 2:**
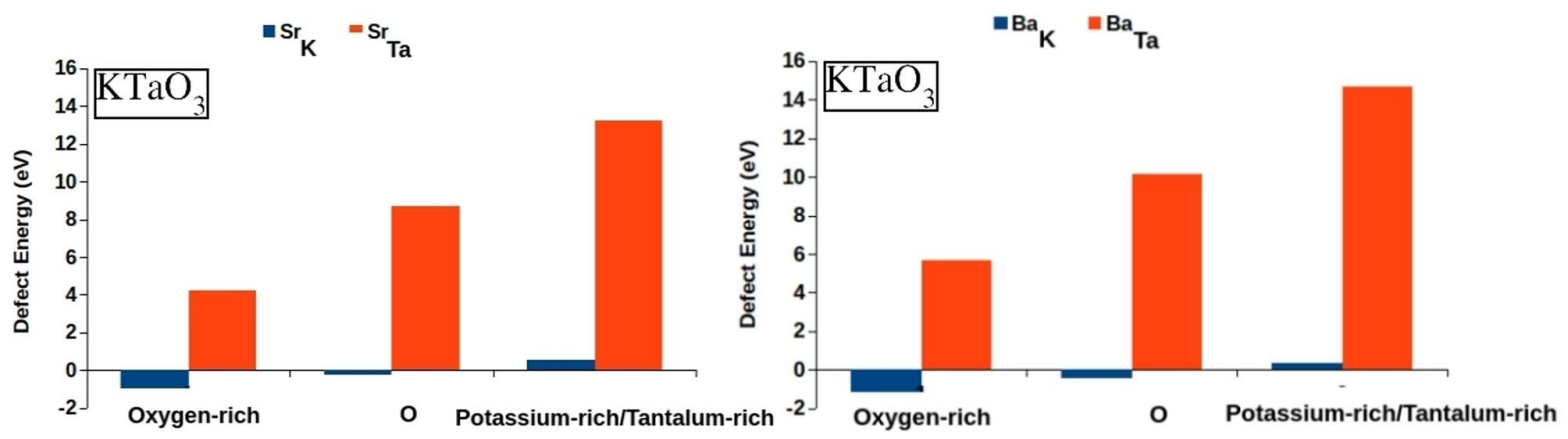
Calculated Defect formation energies for Sr- and Ba-doped KTaO_3_ .

**Fig. 3:**
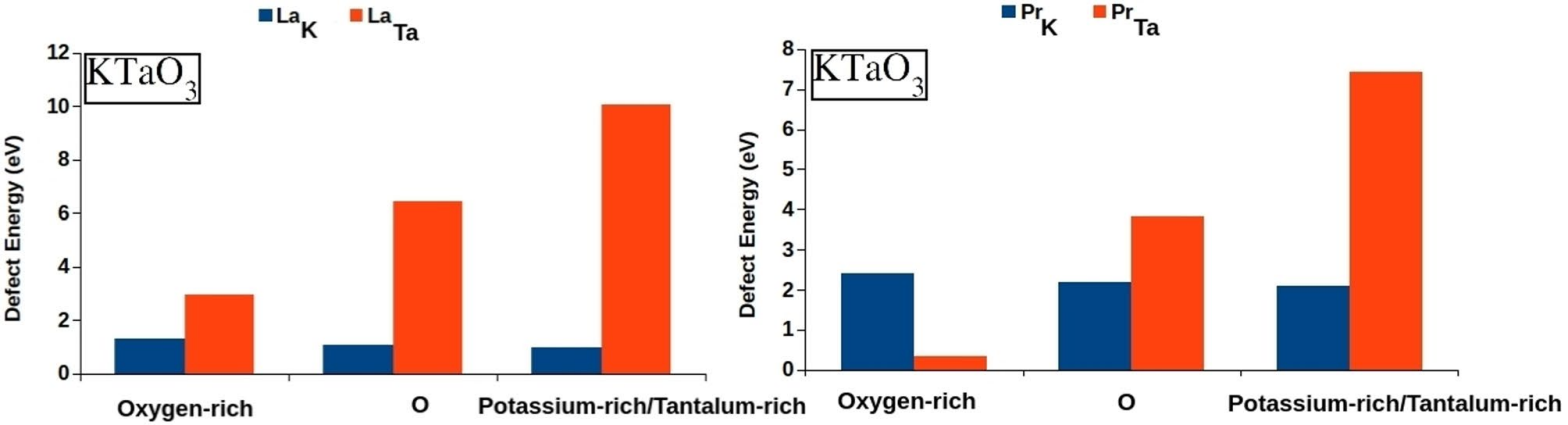
Calculated Defect formation energies for La- and Pr-doped KTaO_3_ .

The enthalpies of formation computed using SCAN functional for SrO, BaO, La_2_O_3_ and Pr_2_O_3_ are−6.056 eV/f.u.,−5.597 eV/f.u.,−17.274 eV/f.u. and−19.157 eV/f.u., respectively, that were used in above equations along with atomic chemical potentials of oxygen shown in Fig. 1 to obtain *Δµ*_*Sr*_, *Δµ*_Ba_ *Δµ*_*La*_ and *Δµ*_*Pr*_ for each stability point. Since two doping sites were to be tested in this work, the dopant at K- or Ta-site of KTO are represented by X_K_ and X_Ta_ respectively. For these two cases, the defect formation energies were computed using the expression^51^.

8 $$\:{E}_{f}\left[{X}_{K/Ta}\right]={E}^{d}-{E}^{p}+{\mu\:}_{K/Ta}\--{\mu\:}_{X}$$

where E^d^ and E^p^ are the total energies of doped and pristine KTO, while *µ*_*K/Ta*_ and *µ*_*X*_ represent the total chemical potentials of the atomic species involved in the substitutional doping process, which vary according to the conditions defined in Eqs. (1)-(7). The calculated defect formation energies for alkaline-earth and rare-earth metals doped KTO systems are presented in the Figs. 2 and 3, which allow us to draw the following conclusions. (i) For oxygen-rich condition, the formation energies of Sr_K_, Ba_K_ and La_K_ dopants in KTO are less than that of the Sr_Ta_, Ba_Ta_ and La_Ta_, respectively, implying that it is thermodynamically more favorable for Sr, Ba and La dopants to occupy the K-site. Under this condition, the defect formation energy of Pr_Ta_ is much lower than that of the Pr_K_ system, indicating that it is more thermodynamically feasible for the Pr dopant to occupy the Ta-site under this condition. (ii) For potassium-rich/tantalum-rich condition, the formation energy of all Sr_Ta_, Ba_Ta_, La_Ta_ and Pr_K_ dopants is lower than X_Ta_, implying that it is thermodynamically more favorable for all the dopant atoms to occupy the K-site. (iii) At point O, the behaviour is similar to that of (ii). Overall, our results indicate that stable incorporation of cation dopants is thermodynamically most feasible under oxygen-rich condition^14,17,18,52–55^. This means that Sr_K_, Ba_K_, La_K_ and Pr_Ta_ are the most stable substitutional dopants in KTO for which we examine the structural, electronic and optical properties in more details. Since the present study focuses on successful substitutional doping of Sr, Ba, La and Pr dopants without presence of competing defects, the results discussed in this work correspond to the situation where physical properties originating from the introduction of these cation dopants in KTO are not influenced by charge compensation.

### Structural properties

The optimization of lattice parameters and internal geometries for pristine, Sr_K_, Ba_K_, La_K_ and Pr_Ta_-doped KTO was carried out using the SCAN meta-GGA functional^42^. Potassium tantalate is a prototype cubic perovskite material crystallizing in the Pm−3 m space group with an experimental lattice parameter of 3.988 Å^48^. The calculated bond lengths between nearest neighbour atoms and the lattice parameter for all modifications of KTO are given in Table 1. Our optimized value of lattice parameter for pristine KTO is in the excellent agreement with available experimental data^48^. Moreover, the lattice parameter predicted by the SCAN meta-GGA functional in this work also shows insignificant divergence in the structural properties compared to those predicted by semi-local functionals (e.g. 4.001 Å reported in Ref^49^. This further strengthens the reliability of the computational approach adopted in the present study. From Table 1, it is evident that pristine KTO has a cubic structure where optimized bond length of K–O and Ta–O are 2.818 Å and 1.993 Å, respectively.

**Table 1:** Lattice parameter, *a*_*0*_, bond lengths, K–O, Ta–O and X-O (where X = Sr, Ba, La and Pr), bond angle near the dopant site, O–Ta–O, and octahedra tilt angle, *α*, for pristine, Sr_K_, Ba_K_, La_K_ and Pr_Ta_-doped KTaO_3_ computed using the SCAN meta-GGA functional.

System	a_0_ (Å)	Bond Lengths (Å) (where X = Sr, Ba, La and Pr)			Bond angle O–Ta–O (degrees)	Octahedra tilt α (degrees)
		K–O	Ta–O	X-O		
Pristine	3.986	2.818	1.993	–	180.000^0^	0.000^0^
Sr_K_-doped	3.970	2.807	1.993	2.759	177.998^0^	0.695^0^
Ba_K_-doped	3.979	2.813	2.003	2.799	178.610^0^	0.914^0^
La_K_-doped	3.973	2.810	2.000	2.720	176.216^0^	1.624^0^
Pr_Ta_-doped	4.055	2.838	1.851	2.203	180.000^0^	0.000^0^

The thermodynamically stable Sr_K_, Ba_K_ and La_K_-doped KTO systems have been modeled by replacing one of the K (potassium) atom with a strontium, barium and lanthanum atoms, respectively in the 2 × 2 × 2 supercell. It can be clearly seen from Table 1 that the lattice parameter of potassium tantalate decreases with the doping of Sr atom, which is in accordance with the larger ionic radius of K^+ 1^ (1.64 Å) in a 12-fold coordination environment compared to that of Sr^+ 2^ (1.44 Å)^56^. The calculated bond lengths after the minimization of internal forces show that the K–O bond length decreases and the Ta–O bond length remains unchanged with respect to pristine KTO upon the introduction of Sr at the K-site of KTO. Moreover, the Sr-O bond length is slightly smaller than the K–O bond length. For the Ba_K_-doped KTO the lattice parameter also decreases due to smaller ionic radius of Ba (1.61 Å)^56^. From the bond length values provided in Table 1, we note that the K–O bond length decreases due to Ba doping. On the other hand, the Ta–O bond length was found to only slightly increase with respect to pristine KTO. Similarly, the lattice parameter was found to decrease upon La doping at the K-site of KTO. This is again in accordance with the smaller ionic radius of the La element (1.36 Å) compared to potassium (1.64 Å) in a 12-fold coordination^56^. The optimized La-O bond length was found to be quite smaller than the K–O bond length. It is evident from Table 1 that the reduction in the crystal volume of Sr_K_, Ba_K_ and La_K_-doped KTO only slightly impact the K–O and Ta–O bond length with respect to pristine KTO. To compensate for the reduction in crystal volume and to keep the Ta–O bond lengths same throughout the supercell, the O–Ta–O bond angles are reduced from their ideal value of 180^0^. This results in an in-phase octahedra tilting, *α*^57^, which attains a maximum value of 1.624^0^ for the case of La_K_-doped KTO. However, the small values of *α* together with the Goldschmidt tolerance factor of Sr_K_, Ba_K_ and La_K_-doped KTO perovskites (discussed below) indicate that a structural transition may not occur in these system under ambient conditions.

The structural properties of Pr_Ta_-doped KTO were also obtained with a 2 × 2 × 2 supercell where a Ta atom was replaced with a Pr atom. Contrary to the decreasing trends in lattice parameter for the other doped systems discussed above, the lattice parameter of Pr_Ta_-doped KTO increases in comparison with the lattice parameter of pristine KTO. The computed Pr-O (2.203 Å) bond length is larger than the nearby Ta–O (1.851 Å) bond length, in accordance with the larger ionic radius of the Pr element (0.99 Å) compared to Ta (0.64 Å) in a 6-fold oxygen coordination^56^. It is interesting to note that the O–Ta–O bond angles within the supercell do not change with the introduction of Pr at the Ta-site of KTO. Since the ionic radii of Pr is larger than Ta, introduction of Pr at the Ta site of KTO causes the lattice volume to expand such that the average value of the Ta–O and Pr-O bond lengths (i.e. 2.027 Å) equals $\:{a}_{0}^{doped}$/2 in Pr_Ta_-doped KTO. Owing to this volume expansion, the buckling of the O–Ta–O bond angle is avoided, which was the main reason behind the octahedra tilts observed in Sr_K_-, Ba_K_- and La_K_-doped KTO^58^. Therefore, octahedra tilting is suppressed in Pr_Ta_-doped KTO (i.e. *α =* 0.000^0^), which ensure that cubic-to-tetragonal phase transitions is not permissible in this system^58^.

In order to examine the structural stability upon introducing Sr_Ta_, Ba_Ta_, La_Ta_ and Pr_K_ dopants in KTO, we have computed the lattice strains using the relationship.

9 $$\:\epsilon\:=\frac{{a}_{0}^{doped}-{a}_{0}^{pristine}}{{a}_{0}^{pristine}}\times\:100$$

where lattice parameters of pristine ($\:{a}_{0}^{pristine}$) and doped ($\:{a}_{0}^{doped}$) systems listed in Table 1 are used. The lattice strains for Sr_Ta_, Ba_Ta_, La_Ta_ and Pr_K_-doped KTO systems were found to be−0.401%,−0.176%,−0.326% and 1.731%, respectively. Since absolute values of ε for all the doped systems are small, lattice strain caused by the substitutional doping of Sr, Ba, La and Pr in KTO lattice is not expected to cause a structural transition^58^. Furthermore, the Goldschmidt tolerance factor of the perovskite lattice for pristine and doped KTO are also computed using^59^.

10 $$\:t=\frac{{R}_{A}+{R}_{O}}{\sqrt{2}\left({R}_{B}+{R}_{O}\right)}$$

where *R*_*A*_ and *R*_*B*_ are the average ionic radii of the atomic species occupying K- and Ta-sites, respectively, in the supercell and *R*_*O*_ is the ionic radii of oxygen (1.40 Å). Compared to the value of tolerance factor of pristine KTO (1.054), the Goldschmidt tolerance factor of Sr_K_, Ba_K_, La_K_ and Pr_Ta_-doped KTO were found to be 1.045, 1.052, 1.042 and 1.032, respectively. Since the tolerance factors of all the doped systems are closer to 1, introduction of these substitutional dopants further stabilizes the cubic phase of KTO^60^.

### Electronic properties

Using the lattice parameters predicted by the SCAN meta-GGA functional, we have computed the electronic structures of pristine and Sr_K_, Ba_K_, La_K_ and Pr_Ta_-doped KTO. Recent DFT study on the alkali-metal tantalates clearly demonstrates that SCAN meta-GGA functional underestimates the energy band gaps of pristine KTO^39^. To overcome this issue, we have employed the mBJ-LDA functional on top of the SCAN optimized structural properties to compute the electronic structure of pristine KTO. The electronic properties predicted by mBJ-LDA functional show good agreement with the experimental data^29,60,61^. Specifically, the indirect (3.582 eV) and direct/optical (4.266 eV) band gaps of KTO computed using mBJ-LDA functional in this work are as close to experimental data^29^ as the ones predicted by HSE06 calculations^61^. This shows that mBJ-LDA facilitates accurate and reliable predictions of electronic and optical properties involving supercell models and significantly cuts the computational costs associated with advanced methods such as hybrid-DFT calculations^62^. Therefore, we only present the electronic, optical and photocatalytic properties computed using mBJ-LDA potential functional to evaluate the photocatalytic performance of the Sr_K_, Ba_K_, La_K_ and Pr_Ta_-doped KTO systems.

**Fig. 4:**
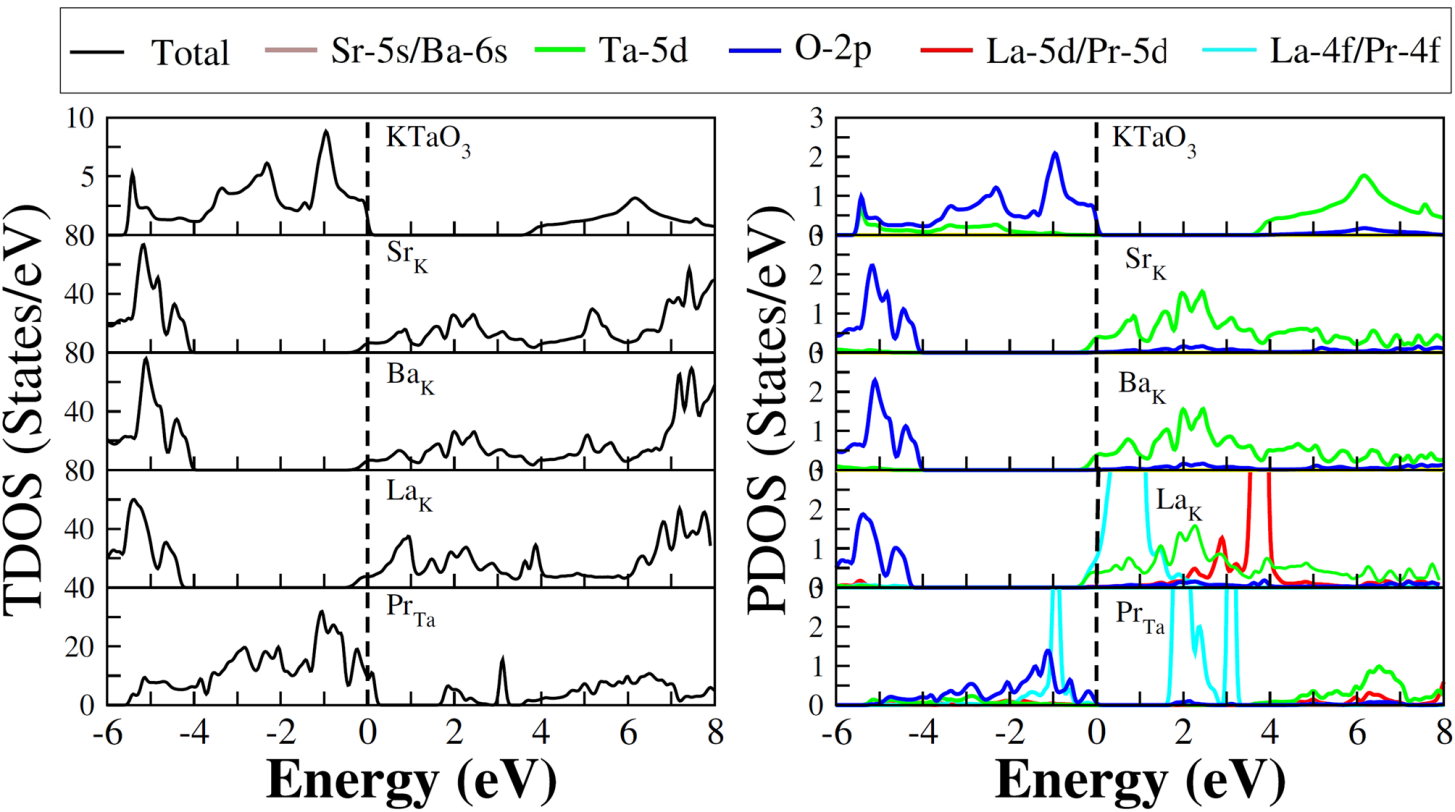
The calculated total density of states (TDOS) and partial density of states (PDOS) plots for pristine and Sr_K_, Ba_K_, La_K_ and Pr_Ta_-doped KTaO_3_ computed using mBJ-LDA meta-GGA functional.

The calculated total density of states (TDOS) and partial density of states (PDOS) plots of pristine and doped KTO are shown in Fig. 4. For Sr_K_, Ba_K_ and La_K_-doped KTO systems, Fig. 4 clearly shows an electron-doped (*n*-type) semiconducting nature. On the other hand, the electronic DOS plots of Pr_Ta_-doped KTO exhibits a hole-doped (*p*-type) semiconducting nature. In case of Sr_K_-doped and Ba_K_-doped KTO, the formal + 2 charge states of Sr and Ba in oxides may be responsible for introducing an extra electron in the KTO. Since these electrons do not take part in the bonding process, they occupy the conduction band states and give rise to *n*-type nature of electronic structure. To evaluate the charge states of these dopant atoms, we compare their effective Bader charges^63^ with K (0.885 *e*) and Ta (3.421 *e*) in pristine KTO. The effective Bader charges of Sr and Ba were found to be 1.713 *e* and 1.787 *e*, respectively, which are in agreement with their + 2 charge in their respective oxides. This is also confirmed from the DOS presented in Fig. 4, where Fermi energy is positioned inside the conduction band. However, for both these systems one can see that the valence band and conduction band retain the predominant contribution of O-*2p* and Ta-*5d* states. Since lower Ta-*5d* states of the conduction band are filled, Pauli exclusion principle requires that electrons from the valence band composed of O-*2p* states could transition to unoccupied Ta-*5d* states above the Fermi energy. The energy gap between the unoccupied O-*2p* and occupied Ta-*5d* states in Sr_K_-doped and Ba_K_-doped KTO were found to be 4.410 eV and 4.359 eV, respectively, which are both larger than the optical band gap of pristine KTO. This indicates that Sr_K_-doped and Ba_K_-doped KTO would exhibit apparent (optical) band gaps larger than pristine KTO’s band gap in accordance with the Burstein-Moss shift^64,65^.

The density of states of La_K_-doped KTO also reveals that the Fermi energy is shifted into the conduction band for this system, such that it also exhibits an *n*-type character. The effective Bader charge of La in La_K_-doped KTO is found to be 2.200 *e*, which is also larger than the effective Bader charge of K in pristine KTO. This suggests that La adopts a + 3 charge state when doped at the K-site of KTO and introduces two extra electrons into the system. This is evident from the DOS plots shown in Fig. 4 where apparent (optical) band gap for La_K_-doped KTO is shown to be ~ 4.688 eV. Because optical band gaps increase with increasing carrier concentration in the conduction band, the apparent band gap of La_K_-doped KTO is clearly larger than the apparent band gaps of Sr_K_ and Ba_K_-doped KTO. Figure 4 shows that the CBM of La_K_-doped KTO is dominated by the La-*5d* and La-*4f* states. While the continuum conduction and valence band edges of La_K_-doped KTO are still primarily composed of Ta-*5d* and O-*2p* states (see Fig. 4), the La-5*d* states hybridize with the Ta-*5d* states of KTO above the Fermi energy. On the other hand, the unoccupied La-*4f* states are found to exhibit sharp and narrow peak at energies where Ta-*5d* states dominate. This shows that La-*4f* states have a localized character^40^, the extra electrons introduced by La in KTO mainly reside in the La-*4f* and Ta-*5d* states below the Fermi energy. Our results are in excellent agreement with earlier studies where alkaline-earth and La-doped KTO have been shown to exhibit *n*-type character^37,44,46,47^.

Finally, replacing one Ta atom with Pr atom makes Pr_Ta_-doped KTO a *p*-type material. Despite having partially filled *4f* states with *4f*^*3*^ *6s*^*2*^ electronic configuration (i.e. 5 valence electrons), the preferred oxidation state of Pr within an oxygen octahedral coordination is usually + 3 or a mixture of + 3 and + 4 as in Pr_2_O_3_ and Pr_6_O_11_^66^, respectively. In Pr_Ta_-doped KTO, the effective Bader charge of Pr is found to be 2.462 e and the Pr-O (2.203 Å) bond length is longer than the Ta–O (2.054 Å) bond length. Both the computed effective Bader charge of Pr and Pr-O bond length are consistent with + 3 charge state of praseodymium^66^. This indicates that not all the 5 electrons of Pr are used for bonding with neighbouring oxygen atoms. Consequently, the *4f* states of Pr dopant split into occupied and unoccupied states that contribute in the valence and conduction bands of KTO, respectively. As evident from Fig. 4, the VBM is mostly composed of O-*2p* states. However, the occupied Pr-*4f* states also appear within the valence band just below the occupied O-*2p* state. Owing to the retention of electron in the *4f* orbitals of Pr, some O-*2p* states become unoccupied and are pushed above the Fermi energy, leading this system to a *p*-type character. On the other hand, the first unoccupied energy band above the Fermi energy (centered around 2 eV) in Pr_Ta_-doped KTO clearly show a Pr-*4f* character. The Ta-*5d* states only appear above 3 eV and show weak hybridization with Pr-*5d* states at higher energies. Since unoccupied Pr-*4f* states below the unoccupied Ta-*5d* states in Pr_Ta_-doped KTO give rise to mid gap states, the apparent band gap for this system is reduced to 2.243 eV compared to pristine KTO. It is worth pointing out here that we have employed spin-polarized calculations to examine the effects of magnetic ordering on the electronic properties of Pr_Ta_-doped KTO^67^. Our results indicate that both spin-polarized and non-spin-polarized calculations lead Pr_Ta_-doped KTO system to a non-magnetic state.

### Optical properties and photocatalytic performance

**Fig. 5:**
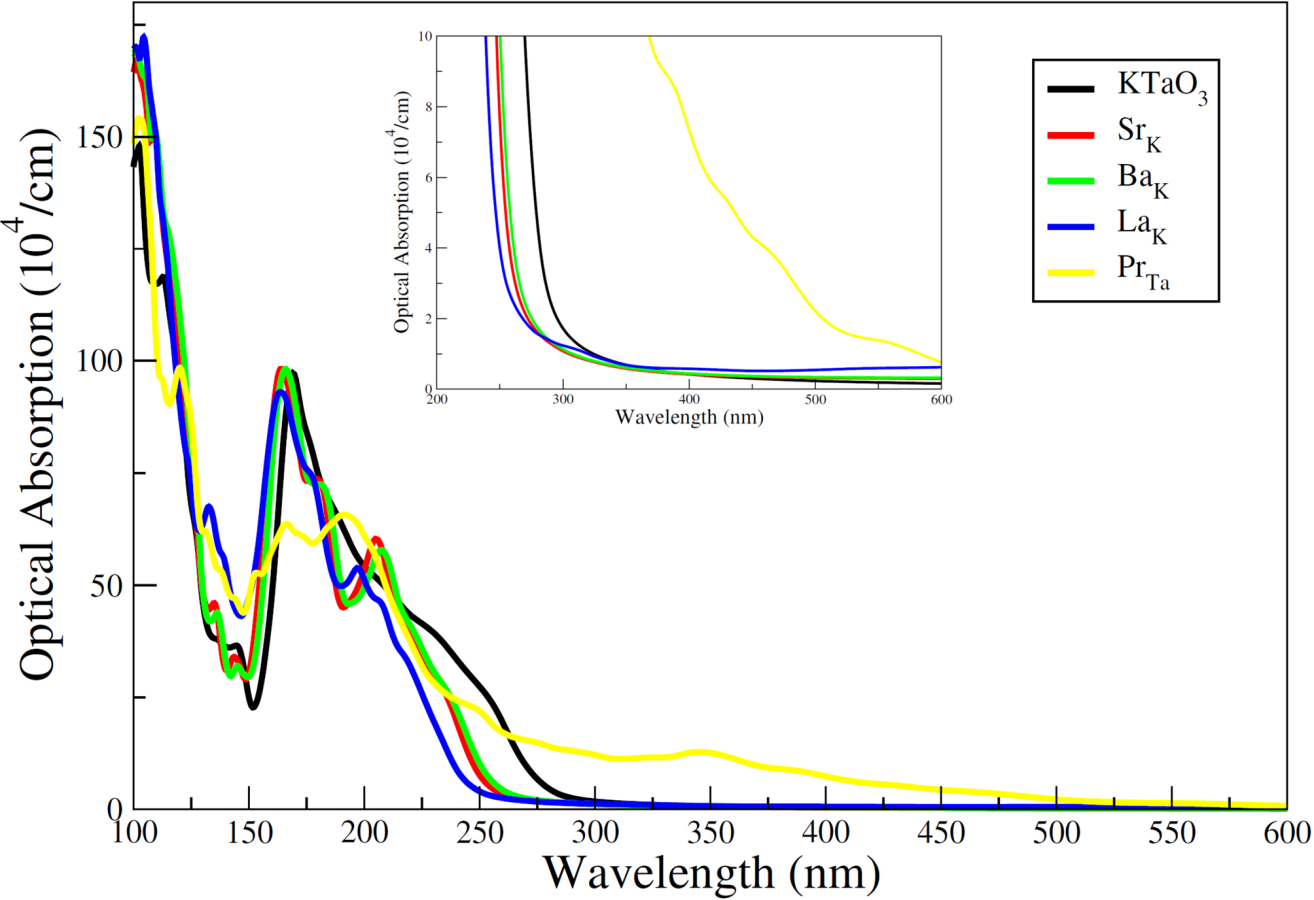
Optical absorption coefficients of pristine, Sr_K_, Ba_K_, La_K_ and Pr_Ta_-doped KTaO_3_ computed using the mBJ-LDA potential functional.

The electronic properties of pristine, Sr_K_, Ba_K_, La_K_ and Pr_Ta_-doped KTO demonstrate that the origins of the optical band gaps of these systems are significantly different. These results are consistent with earlier studies which demonstrates that the presence of vacancy^68,69^ or dopant^70–72^ can significantly alter the energy gaps and optical properties of semiconductors. Since optical absorption is an important parameter to examine the applications of a crystalline material, it is pertinent to examine the optical properties of pristine and doped modifications of KTO studied in this work. For photocatalytic application potential, we are mainly interested in the impact of Sr, Ba, La and Pr dopants on the light absorption ability of KTO. For this reason, the optical absorption coefficients for pristine and doped KTO were computed that are show in Fig. 5.

From the computed optical absorption coefficients shown in Fig. 5, it can be observed that in case of pristine KTO the absorption ability is limited to the UV region (photons with wavelength < 290 nm) of the electromagnetic spectrum. This result is in accordance with experimental observations^24,26,49^. Interestingly, only the optical absorption coefficients of Pr_Ta_-doped KTO are found to have values > 1 × 10^4^ /cm for photons having wavelengths less than 500 nm (see inset Fig. 5). This is due to emergence of mid gap composed of localized Pr−4*f* states in this system that allow weak absorption of visible light in Pr_Ta_-doped KTO and potentially enhance its solar active photocatalytic potential^71,72^. It is also evident from Fig. 5 that doping of Sr, Ba and La at K-site of KTO shifts the absorption edge to ~ 281 nm, ~ 284 nm and ~ 264 nm, respectively. Therefore, as a result of *n*-type doping, the onset of optical absorption of photons having wavelength < 280 nm indicates the blue shift of the apparent optical band gap in these doped systems^64,65^. For the Sr_K_-, Ba_K_- and La_K_-doped systems, we have also computed transition-resolved optical absorption coefficients for transitions from occupied electronic states (between −1 eV and Fermi energy) to unoccupied electronic states above Fermi energy, as shown in Fig. 4. These computations clearly show that intra-band optical transitions do not contribute to the main optical absorption coefficient peaks of Sr_K_, Ba_K_ and La_K_-doped KTO shown in Fig. 5. This is understandable because the optical transitions from the occupied states just below the Fermi energy to the unoccupied states of conduction bands in these *n*-type systems are forbidden owing to the dominance of Ta−5*d* states above the Fermi energy. The observed shift in the onset of optical absorption together with the availability of charge carriers in the conduction band of Sr_K_, Ba_K_ and La_K_-doped KTO due to *n*-type doping suggests that these system may exhibit better photocatalytic response under UV irradiation^24,26,49,71^.

Due to its large band gap, the CBM and VBM of pristine KTO are suitably positioned above and below, respectively, from the redox potentials of H^+^/H_2_ and H_2_O/O_2_ for water splitting process^15^. While we only find Pr_Ta_-doped KTO suitable for visible light absorption, the presence of charge carriers in the conduction band of Sr_K_, Ba_K_ and La_K_-doped KTO together with their slightly blue shifted optical absorption coefficients may also be suitable for photocatalytic applications under UV irradiation. In order to examine the photocatalytic potential of pristine, Sr_K_, Ba_K_, La_K_, and Pr_Ta_-doped KTO, we compare the band-edge potentials of CBM and VBM using the formulas^71,73^

11 $$\:{E}_{CBM}={\chi\:}^{GM}+{E}_{0}\--\frac{{E}_{g}^{op}}{2}$$

and

12 $$\:{E}_{VBM}={\chi\:}^{GM}+{E}_{0}+\frac{{E}_{g}^{op}}{2}$$

**Fig. 6:**
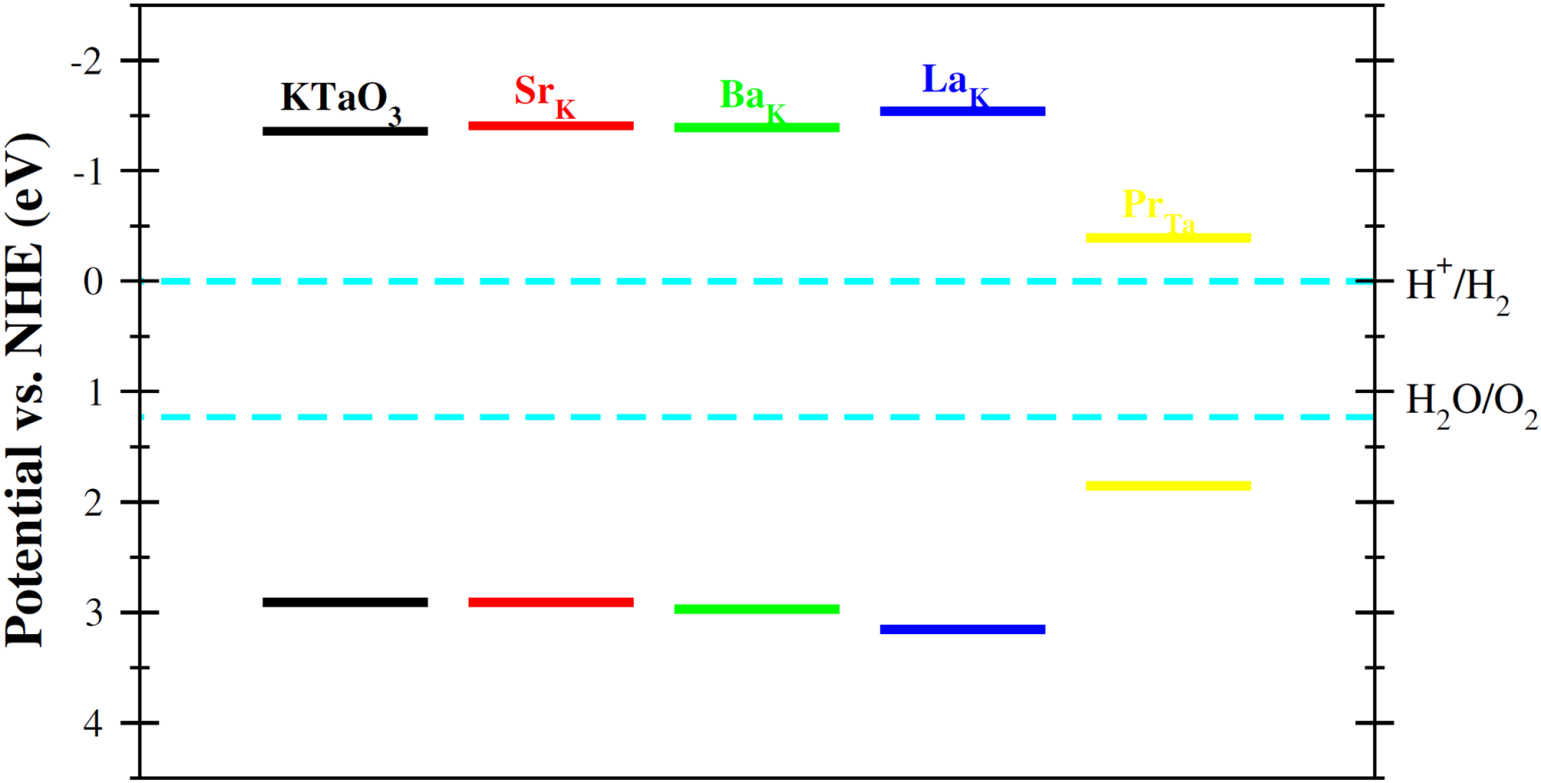
The computed band-edge potentials vs. normal hydrogen electrode potentials for pristine, Sr_K_, Ba_K_, La_K_, and Pr_Ta_-doped KTaO_3_. The redox potentials of H^+^/H_2_ and H_2_O/O_2_ are represented by dashed cyan line.

In Eqs. (11) and (12), *E*_*g*_^*op*^ represents the direct band gap of pristine KTO, direct apparent band gap up to mid gap state of Pr_Ta_-doped KTO and the apparent band gaps of Sr_K_, Ba_K_ and La_K_-doped KTO. *E*_*0*_ is the normal hydrogen electrode potential (=−4.5 eV)^74^, whereas $\:{\chi\:}^{GM}$ represents the geometric mean of Mulliken electronegativities of the atoms in the supercell^75^. The geometric mean of Mulliken electronegativities for pristine KTO is 5.276. Comparison of this value with the geometric mean of Mulliken electronegativities for pristine SrTiO_3_ (~ 5.317) clearly indicates that KTO’s CBM has a higher reduction ability and better performance in driving hydrogen evolution reaction^15^. Upon introducing dopant into KTO lattice, the geometric mean of Mulliken electronegativities are found to be 5.298,5.289, 5.307 and 5.230 for Sr_K_, Ba_K_, La_K_, and Pr_Ta_-doped KTO, respectively. Since none of the doped system attains $\:{\chi\:}^{GM}$ greater than that of SrTiO_3_, the good reduction ability of KTO in driving hydrogen evolution reaction is not expected to be severely impacted by introduction of the dopants considered in this work. In addition to availability of charge carriers in the conduction band of Sr_K_, Ba_K_ and La_K_-doped KTO due to *n*-type doping, the appropriate positioning of their band edge potentials shown in Fig. 6 supports their photocatalytic hydrogen production potentials observed in experiments^36,37,44^. On the other hand, Pr_Ta_-doped KTO with smallest value of $\:{\chi\:}^{GM}$, better absorption of visible and UV light and CBM and VBM positioned closer to the redox potentials (see Fig. 6) indicate that praseodymium doping in KTO can be beneficial for hydrogen production via solar active photocatalysis.

## Conclusions

In conclusion, the effects of alkaline-earth metals (Ba and Sr) and rare-earth (La and Pr) dopants on the structural, energetic, electronic and optical properties of KTO have been investigated by employing first-principles DFT calculations. The structural properties were calculated using the SCAN meta-GGA functional, which showed excellent accord with experimental data. The optimized lattice parameter of the Sr_K_-doped, Ba_K_-doped and La_K_-doped KTO systems decreased compared to pristine KTO, which can mainly be attributed to the smaller ionic radii of Sr ^+ 2^ (1.44 Å), Ba ^+ 2^ (1.61 Å) and La ^+ 3^ (1.36 Å) compared to that of K ^+ 1^ (1.64 Å) in a 12-fold coordination environment. On the other hand, introducing a Pr atom at the Ta-site of KTO causes the lattice parameter to increase. The Pr-O bond length (2.203 Å) and effective Bader charge of Pr (2.462 *e*) indicate that praseodymium adopts a + 3 charge state in Pr_Ta_-doped KTO. The calculated defect formation energies, within the allowed chemical potentials limits of the atoms involved in the doping process, indicate that under an oxygen-rich chemical condition Sr_K_, Ba_K_, La_K_ and Pr_Ta_ are the most stable dopants in KTO. This is promising because cation dopants in perovskite oxide without the presence of oxygen vacancies are generally good photocatalysts. Our results reveal that Sr_K_, Ba_K_ and La_K_-doped KTO are *n*-type semiconductors due to the shift of Fermi energy into the conduction band. In the case of Sr_K_-doped and Ba_K_-doped KTO, the valence band and conduction bands are found to retain their O−2*p* and Ta−5*d* characters, respectively. On the other hand, the conduction band of La_K_-doped KTO was found to be predominantly composed of Ta−5*d* along with contributions coming from La−4*f* and La−3*d* states. For the Sr_K_, Ba_K_ and La_K_-doped KTO systems, the apparent band gap were found to be larger than pristine KTO. Despite its *p*-type nature, the optical absorption of Pr_Ta_-doped KTO was found to improve in the visible region of the electromagnetic spectrum. The emergence of *p*-type nature was found to be due to the presence of occupied *4f* states of Pr in the valence band. The reduced band gap of Pr_Ta_-doped KTO is due to the mid gap states composed of unoccupied Pr−4*f* states. Our results show that the variation in the physical properties from doping of Sr, Ba, La and Pr in KTO may be useful in designing photocatalysts with improved hydrogen production performance.

## Data Availability

The datasets used and/or analysed during the current study available from the corresponding author on reasonable request.
